# Latency-Sensitive Function Placement among Heterogeneous Nodes in Serverless Computing

**DOI:** 10.3390/s24134195

**Published:** 2024-06-27

**Authors:** Urooba Shahid, Ghufran Ahmed, Shahbaz Siddiqui, Junaid Shuja, Abdullateef Oluwagbemiga Balogun

**Affiliations:** 1 Department of Computer Science, National University of Computer and Emerging Sciences, Karachi 75030, Pakistanghufran.ahmed@nu.edu.pk (G.A.); shahbaz.siddiqui@nu.edu.pk (S.S.); 2 Department of Computer Science, COMSATS University Islamabad, Abbottabad Campus, Abbottabad 22060, Pakistan; 3 Department of Computer and Information Sciences, Universiti Teknologi PETRONAS, Seri Iskandar 32610, Malaysia; abdullateef.ob@utp.edu.my

**Keywords:** server less, multi-tier, distributed, hierarchical, cloud, edge, machine learning

## Abstract

Function as a Service (FaaS) is highly beneficial to smart city infrastructure due to its flexibility, efficiency, and adaptability, specifically for integration in the digital landscape. FaaS has serverless setup, which means that an organization no longer has to worry about specific infrastructure management tasks; the developers can focus on how to deploy and create code efficiently. Since FaaS aligns well with the IoT, it easily integrates with IoT devices, thereby making it possible to perform event-based actions and real-time computations. In our research, we offer an exclusive likelihood-based model of adaptive machine learning for identifying the right place of function. We employ the XGBoost regressor to estimate the execution time for each function and utilize the decision tree regressor to predict network latency. By encompassing factors like network delay, arrival computation, and emphasis on resources, the machine learning model eases the selection process of a placement. In replication, we use Docker containers, focusing on serverless node type, serverless node variety, function location, deadlines, and edge-cloud topology. Thus, the primary objectives are to address deadlines and enhance the use of any resource, and from this, we can see that effective utilization of resources leads to enhanced deadline compliance.

## 1. Introduction

**Figure 1:**
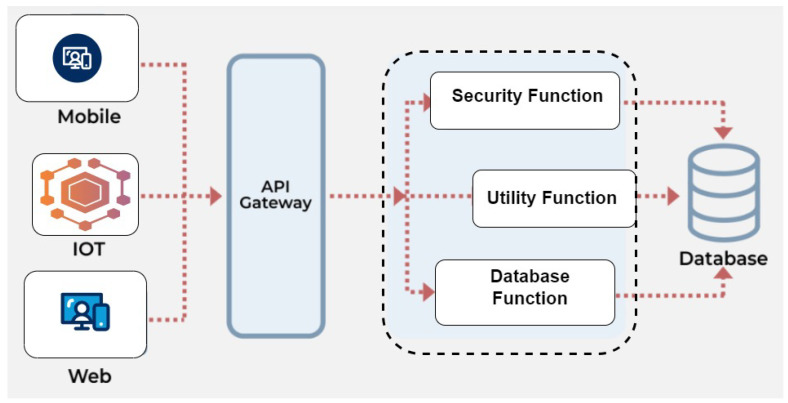
An example of serverless application.

In the contemporary cloud infrastructure landscape, a hierarchical multi-tier design has become essential, seamlessly connecting remote edge devices, local edge resources, and the central cloud network. This design significantly reduces latency for remote-edge devices, enhancing computational capabilities [1]. Amidst this evolution, the serverless model, or Function as a Service (FaaS) [2], has emerged as a game-changer. It liberates businesses from server and resource management, allowing them to focus solely on their core business logic and application operation [3]. The serverless model shifts server and resource management responsibility to the cloud provider, with developers deploying code and ensuring essential functions for execution [4]. The shift towards FaaS brings opportunities and challenges, fundamentally changing how businesses and developers interact with cloud infrastructure [5]. In the realm of infrastructure as a service (IaaS), developers can harness the network, computation, and storage resources provided by cloud service providers like Amazon EC2 instances and S3 buckets. However, it does not shield developers from the intricacies of operating these resources, leaving them responsible for code management and provisioning. In stark contrast, serverless computing completely conceals the underlying infrastructure of servers and resource management while bestowing complete control of the application upon developers [6,7,8]. The serverless architecture not only ensures scalability but also slashes operational costs. It is available on both private and public serverless platforms, with notable examples including AWS Lambda [9] and Azure Functions [10], offered by public cloud service providers. Software developers can dynamically add containers to these platforms to scale applications according to their policies. Events such as user requests to the function’s URL typically trigger the functions hosted within these containers. When multiple requests overwhelm the system, the system automatically scales up the containers to meet the surge in demand. When a function is invoked for the first time, the cloud creates a container for it, a process known as a cold start [11,12,13,14]. The cloud provider either stops or pauses containers during periods of inactivity. When a new request is received, the cloud initiates or reactivates existing containers (or spawns new ones if none are available or all are engaged with other requests), commonly referred to as a warm start [5]. Warm starts exhibit lower latency than cold starts, enhancing overall performance [11,15]. In the serverless architecture, functions are treated as discrete, independently scalable services, which is visually represented in Figure 1. This figure illustrates a typical serverless setup where different types of clients such as mobile applications, IoT devices, and web interfaces interact with the serverless platform through an API Gateway. This gateway serves as the entry point for all client requests and directs them to the appropriate function within the serverless environment.

The Security Function [16], as depicted, is responsible for authenticating and authorizing requests, ensuring that only valid and permitted actions are executed. The Utility Function performs specific operations that may involve logic processing or data transformation, whereas the Database Function handles interactions with the database, such as retrieving or updating records. Events, specifically requests routed through the API Gateway, invoke these event-driven functions.The rise of the Internet of Things (IoT) has propelled the need for low-latency solutions to the forefront [17]. In the context of the highly distributed Cloud Edge system, strategically placing functions to meet user requirements emerges as a critical challenge.

**Figure 2:**
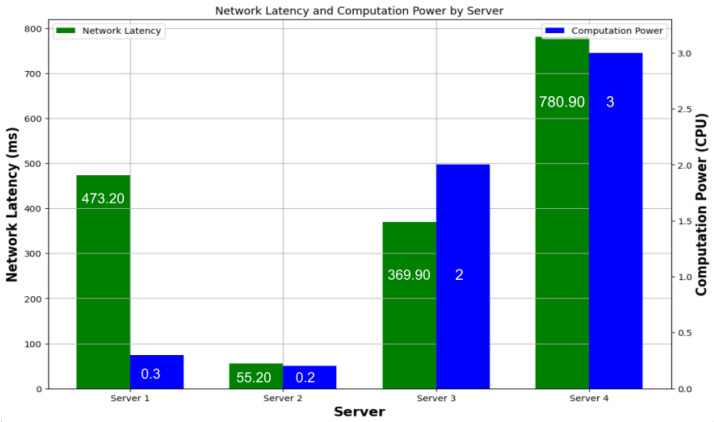
An example of network delay vs. processing time.

Current popular open-source serverless platforms, such as OpenLambda [18], OpenWhisk [19], and OpenFaaS [20], fundamentally lack consideration in two critical areas: (1) the heterogeneity of execution nodes and (2) the alignment of function placement decisions with the deadlines of tasks. This oversight can lead to incorrect function placement decisions, contributing to unpredictability in function execution. Additionally, network latency emerges as a significant factor in these placement decisions. When tasks are associated with specific deadlines, it becomes imperative to accurately determine which execution node best matches the resource requirements of the function and whether the function can fulfill its execution within the given constraints.As workloads are inherently variable, there is a pressing need for tools that can adapt to these fluctuations seamlessly [21,22]. Machine learning stands out as a promising tool in this context, offering the ability to learn from historical data and make informed decisions based on current trends. Despite its potential, none of the existing open-source serverless platforms currently incorporate machine learning as a default feature for optimizing function placement decisions. This gap indicates a significant opportunity for enhancing the efficiency and reliability of serverless computing platforms by integrating machine learning algorithms capable of adapting to changing workloads and optimizing function placement in real time. Consider the scenario where a user, referred to as ‘S’, initiates a video conversion request with an expectation for timely completion. Figure 2 serves as a data-driven guide for optimal function placement within a serverless architecture. The graph’s X-axis categorizes four distinct servers, each with unique computational power, measured in CPU units, which is vital for processing ‘S’ requests. The left Y-axis maps out network latency in milliseconds, a crucial factor considering the stringent ‘S’ deadline. The user sends a request from coordinates (−46.6417, −23.5733), and each server is located at a different physical location, for example, Server1 (Santa Clara), Server2 (Brazil), Server3 (Dallas), Server4 (Hong Kong) [16]. Ultimately, the figure underscores the complexity of function placement in heterogeneous environments. The decision is not about choosing the fastest or the most powerful server but finding the one that aligns computational capabilities with network efficiencies to adhere to the user-defined quality of service (QoS) metrics.

Incorporating machine learning into serverless platforms could enable these systems to dynamically analyze current network conditions, node performance, and workload characteristics to make real-time decisions on function placement. By doing so, serverless architectures can optimize the execution of functions across the heterogeneous landscape of execution nodes, ensuring that each task is executed on the node that offers the best balance of low latency and high processing power to meet its deadline [22,23]. This approach not only mitigates the risk of unpredictability in function execution but also maximizes the efficiency and reliability of serverless computing platforms in handling variable workloads.

## 2. Motivation

The fundamental motivation for this work is to optimize function placement in serverless computing environments while accounting for network latency and achieving deadlines. The following points highlight the key motivations behind this research:

1. Erratic Execution in Open-Source Serverless Solutions: The execution of open-source serverless solutions is often erratic due to their failure to account for the diversity of execution nodes and to synchronize function placement with task deadlines.
2. Impact of Network Latency: Network latency significantly influences decisions regarding function placement, especially in the context of time-sensitive tasks. Assessing which execution node most effectively fulfills the function’s resource demands and determining whether the function can execute within the given constraints is of utmost importance.
3. Need for Adaptable Technologies: To effectively handle unforeseen tasks, adaptable technologies are vital. In this regard, machine learning emerges as a highly promising instrument due to its ability to learn from past data and generate sophisticated assessments based on the most recent patterns.

### Contribution

The author of [24] examines time-sensitive operations in a video surveillance program, such as image resizing, face detection, and video conversion, while accounting for network and processor latency. The goal is to maximize resource consumption and ensure job completion in a serverless architecture using Docker containers for simulation. This study addresses the difficulty of efficiently deploying functions in a hierarchical edge-cloud architecture while meeting deadlines and optimizing resource use. It takes into account the heterogeneity of compute nodes (CPU, RAM, and memory) and uses a linear machine learning model with offline data collection to develop an optimal placement strategy. This work is an extension of the existing framework and includes the following significant contributions:

- The study provides an automated function placement technique using machine learning to optimize function placement in serverless computing environments. The technique employs non-linear machine learning models to predict the execution time and network latency, guaranteeing the completion of functions within the specified time frames and quality of service (QoS) standards.
- The study shows the success of the proposed technique in a real-world video surveillance application. The method involves integrating stateless services into a cloud-based surveillance system to ensure task execution is timely and efficient.
- This study compares the suggested machine learning-driven function placement method against non-ML techniques like Best Fit and Round Robin. The results reveal that the proposed technique beats these algorithms in terms of fulfilling deadlines and ensuring quality service standards.
- The study highlights advancements in automating and improving function deployment in serverless computing systems. The technique can manage complex interactions between input features and output variables by relying on machine learning models, resulting in increased serverless computing performance and efficiency.

The rest of the article is structured as follows. Section 3 presents the literature review on function placement in serverless computing. The proposed research methods for serverless computing and machine learning algorithms are discussed in Section 4. Section 5 presents the proposed methods. The results and evaluation of proposed methods is provided in Section 6. Section 7 provides the conclusion and future research directions.

## 3. Literature Review

The research in [4] proposes a novel approach called AuctionWhisk that addresses function placement in serverless fog platforms using an auction-based model. The paper focuses on the challenges of optimizing the placement of functions in serverless fog environments, where resources are distributed across various edge devices and cloud servers. It introduces AuctionWhisk as a solution to this problem, drawing inspiration from auction mechanisms to efficiently allocate functions to appropriate resources. The authors in [15] investigate a fog computing system consisting of multiple tiers, including edge, intermediary, and cloud nodes, all of which operate within a Function-as-a-Service (FaaS) framework. The primary objective is to improve the process of offloading tasks, allocating resources, and increasing the revenue of service providers. To achieve this, the research adopts an action-based approach, where developers compete by submitting bids for available nodes and execution time. These nodes include edge, intermediary, and cloud nodes, allowing for task forwarding when a node reaches its maximum capacity. Martinez, M.M. et al. [25] have presented a middleware solution that leverages a Kalman filter to distribute requests among different nodes. The design of this approach aims to reduce response times and maintain a balanced computational load. The chosen performance metric of interest is CPU utilization, which serves as an indicator of the system’s activity level. Higher CPU utilization values correspond to longer request processing times. In this setup, nodes share information about their resource usage, encompassing metrics like CPU and memory. In the paper [26], the authors present a heuristic algorithm designed to facilitate the optimal selection of virtual machines (VMs) to meet deadlines and reduce provider costs. To implement this model, the authors employ ContainerCloudSim and conduct simulations. They highlight a gap in previous research that primarily focused on resource optimization and failed to adequately consider the specific requirements of individual applications, potentially leading to breaches of service-level agreement (SLA) requirements. To address the above-mentioned issues, the article introduces two algorithms:

- Deadline Function Placement (DBP): This algorithm prioritizes the placement of functions on the VM with the highest computational capacity. If deadlines are not met, it allows for rescheduling to ensure compliance with the deadlines.
- Dynamic Resource Alteration (DRA): DRA focuses on selecting nodes for functions that were previously evicted to ensure their optimal placement in a manner that aligns with the application’s requirements and performance goals.

Das et al. [27] put forward a strategy for scheduling multi-function applications across both private and public cloud environments, taking into account the importance of task priority and meeting deadlines for batch jobs. The primary goal is to cut costs by optimizing cloud resource selection. The process starts by directing the initial function requests to the private cloud. We conceptualize the application as a directed acyclic graph (DAG) to illustrate the order of execution. The system introduced in [28], known as FADO, is designed for managing data replication and the placement of storage buckets based on user-defined policies. It maintains a database that contains information about replication and storage bucket configurations, as well as user HTTP requests for function invocation. The system employs multiple servers, including an HCP server, an edge server, and a cloud server. Users configure the storage bucket and specify data replication on these servers based on their predefined setups. FADO is capable of updating any changes made in replication on these servers, eliminating the need to copy the entire dataset again in case of modifications. The article [17] presents a swarm-based solution where a global master node is provided with a predefined sequence of functions. The global master node then assigns and places these functions on the Multi-Access Edge Computing (MEC) server for processing. The users subsequently receive the processed results. The author evaluates the system based on metrics such as network latency, resource utilization, and execution time. The proposed mechanism excels in terms of network latency, demonstrating superior performance in this area. A good way to assign containers to functions is described in [29]. It is based on predictions of the workload and is called Lass: latency-sensitive serverless computations at the edge. The system utilizes a model trained with queuing models and two sliding window methods (2 min and 10 s). It initiates container launches once it has identified anticipated resource shortages for functions. Notably, Lass also introduces resource reclamation, recovering excess CPU and memory allocations and implementing a fair share mechanism. With a fair share, users can specify minimum resource needs, guaranteeing fair allocation across functions, thus ensuring equitable resource distribution and mitigating potential over-provisioning. In ref. [30], the paper presents a method that merges a Q-learning algorithm and a scaling policy to optimize the resource configuration of serverless services. The Q-learning algorithm fine-tunes resource allocation to enhance performance while adhering to response time constraints. Meanwhile, the scaling policy combines vertical and horizontal scaling methods to address varying workload requirements. This approach encompasses pre-warmed instances, down-scaling strategies, and a stabilization parameter to ensure seamless transitions. In ref. [7], the paper introduces DeF-DReL, a term coined to introduce their proposed system, which is a serverless function deployment strategy designed for fog and cloud computing settings. The algorithm’s design aims to lessen the workload in the fog environment while simultaneously guaranteeing optimal serverless application performance and compliance with user application deadlines. In ref. [31], the paper presents an innovative combination of serverless and edge computing to improve the efficiency of applications in edge environments. This integration has the potential to result in efficiency improvements, reduced latency, and enhanced real-time performance. This framework could make edge computing a lot better by using serverless resource allocation and the closeness of edge computing. This could be especially useful in industrial IoT settings and could lead to more research in this area. In ref. [32], the paper explores the viability of using serverless platforms for latency-sensitive applications. The research evaluates the suitability of serverless computing for meeting the demands for quick response times, delving into areas such as function deployment, resource allocation, and communication patterns. This study provides valuable insights into the capacity of serverless architectures to fulfill the requirements of time-sensitive applications.

In ref. [33], the paper investigates methods for addressing latency-sensitive applications on public serverless edge cloud platforms. It likely encompasses topics such as resource allocation, communication optimization, and function placement strategies to achieve peak performance in dynamic edge environments. The results are of significance to industries like IoT and multimedia, providing valuable insights for researchers and practitioners looking to improve the deployment of latency-sensitive applications. In ref. [8], the paper delves into the deployment of serverless functions in the cloud-edge continuum. It examines the challenges and opportunities within this hybrid environment, possibly covering topics like function deployment, resource allocation, data handling, and load balancing. The results shed light on the application of serverless computing in distributed systems, presenting valuable insights for consideration in various applications. In summary, the literature review can be summarized as follows in Table 1.

## 4. Machine Learning

Machine learning, a subset of artificial intelligence (AI), involves training algorithms to make data-driven predictions or decisions. It is a type of supervised learning where the algorithm learns from labeled data and then predicts outcomes for new, previously unseen data. Applications of machine learning include image and speech recognition, natural language processing, recommender systems, and predictive modeling. Linear models are a type of machine learning technique that predicts output using a linear combination of input features. They are straightforward and simple to grasp, making them popular for a wide range of applications.

1. Linear regression utilizes tasks with linear regression, a type of linear model. Using one or more input features, it predicts a continuous output variable. The purpose of linear regression is to find the best-fitting line that minimizes the mean squared error between predicted and observed values.
2. Logistic regression is a type of linear model that is used for classification. It predicts a binary output variable (0 or 1) using one or more input features. The purpose of logistic regression is to determine the best-fitting line that divides the classes with the greatest accuracy.

Non-linear models are machine learning algorithms that predict output using a non-linear mix of input features. They are more complicated and powerful than linear models but more difficult to comprehend and interpret.

1. Decision trees are non-linear models that use a tree-like structure to classify or predict data. Each node in the tree represents a feature or a decision, with branches representing potential outcomes. Decision trees are simple to grasp and analyze, but they are susceptible to overfitting.
2. Random forests are an ensemble method that uses several decision trees to increase the model’s accuracy and robustness. We train each tree on a random subset of data and features and aggregate the predictions of all trees to obtain the final prediction.

Function as a Service (FaaS) enables the deployment and operation of both linear and non-linear machine learning models as serverless functions. The cloud provider manages the underlying infrastructure, making it simple to develop and expand machine learning models. FaaS allows developers to quickly deploy and test various machine learning models, including linear and non-linear models, without worrying about the underlying infrastructure. This allows for quick experimentation and iteration, which is critical in the development of machine learning applications. Furthermore, FaaS can help save money by just running machine learning models when they are required, rather than maintaining a dedicated server or cluster.

## 5. Proposed Methodology

**Figure 3:**
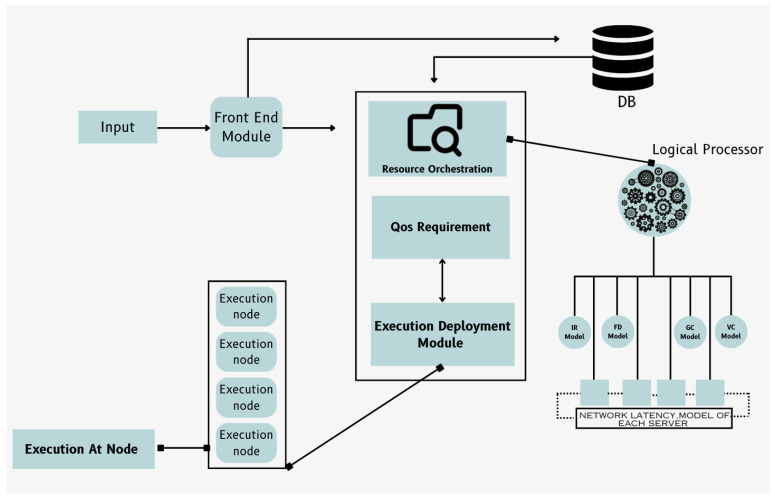
High-level diagram of proposed model.

In this section, the outlined methodology is employed to introduce deployment strategy. The system proposed is developed from the ground up and comprises of various modules. A high-level diagram of the proposed system that encapsulates the whole proposed system can be viewed in Figure 3. This section will delve into these modules, building the proposed architecture while addressing the existing research gaps:

- **Front End Module:** Users interact with this component by submitting their functions through HTTP requests. They provide input data along with the function and the event’s name.
- **Resource Orchestrator:** As the keystone of the proposed system, the resource orchestrator orchestrates the execution of functions across the optimal set of resources. It leverages a sophisticated framework to assess the platform’s capabilities and optimization criteria, ensuring that each function is executed within its specified QoS parameters and deadlines.
- **Execution Node Handler:** This service operates on each worker node within the diverse resource pool of the Function-as-a-Service (FaaS) platform. It manages the lifecycle of functions running on these nodes, ensuring efficient execution.
- **Logic Processor:** Upon receiving each incoming request, the Logic Processor evaluates a range of potential resource configurations for executing a function. It does so based on the user’s specified QoS requirements and network latency between user and heterogeneous node, aiming to meet those requirements effectively.

The workflow initiates when users, through a Flask-implemented HTTP request, submit their multimedia tasks alongside pivotal details such as the function name, input parameters, QoS expectations, and their geographical coordinates. These data are meticulously logged and remain in a MongoDB database for integrity and traceability.

Subsequently, the Resource Orchestrator assimilates the request’s details—encompassing the function name, input specifics, QoS requirements, and user location—channeling this data to the Logic Processor. The Logic Processor, leveraging pre-trained models tailored to the requested function and network latency predictions, meticulously analyzes the execution nodes’ suitability against the task’s deadline and QoS criteria. This analysis culminates in a judicious decision regarding the function’s optimal deployment location, prioritizing edge or cloud nodes based on the feasibility of meeting the deadline effectively. The Execution Deployment module then takes the helm, materializing the Logic Processor’s decision by orchestrating the function’s deployment to the designated location. This strategic placement, whether proximal at the edge or centralized in the cloud, ensures that multimedia processing tasks are executed within the stipulated deadlines, thereby enhancing the system’s responsiveness and reliability.This methodology underscores our commitment to advancing cloud-based multimedia processing technologies, offering a robust, scalable solution that meticulously addresses the evolving needs and expectations of users in a dynamically digital environment.The core components are discussed below.

### 5.1. Logic Processor

The Logic Processor plays a pivotal role in the system’s decision-making process by overseeing the executive nodes. Within this module, each function that enters the system is associated with a specific machine learning model. There are two distinct machine learning models integrated into the logic processor: one for execution time prediction and the other for network latency prediction, as detailed in Algorithms 1 and 2. We train the execution time prediction model using input data like image or video resolution, and the output is the estimated execution time for each function. We train this model using XGBoostRegressor on a dataset of 880 records, logging each function execution for each executive node. Our system is heterogeneous, executing each function across all nodes. For network latency prediction, the model takes longitude and latitude as input to estimate network latency for each executive node. Each executive node maintains a single model, trained on a dataset of 189 records containing various longitude and latitude combinations. We utilize the decision tree regressor algorithm for training. We predict the network latency for each executive node. The user then filters the outputs of both machine learning algorithms against the task’s specified deadline, ensuring timely and efficient task completion. We can summarize the Logic Processor’s workings as follows:

**Algorithm 1:** Execution Time Prediction Algorithm

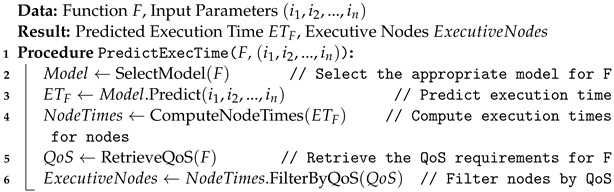



**Algorithm 2:** Network Latency Prediction

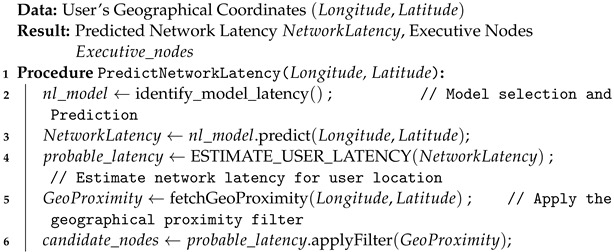

1. **Execution Time Prediction:** This initial stage focuses on estimating the time required to execute the function based on the resolution of the input image or the size of the input video. The prediction utilizes a pre-trained machine learning model, specifically tailored to assess execution time in relation to the unique characteristics of the input data.
  (1) ExecutionTime=f(InputSize,MLmodel)
  where:
  - *InputSize* denotes the resolution of the image or the size of the video.
  - *MLmodel* represents the machine learning model used for execution time prediction.
  - *f* encapsulates the predictive function that calculates the estimated execution time.
2. **Network Latency Prediction:** Subsequent to execution time estimation, this stage evaluates the network latency based on the geographical coordinates of the user. Employing another specialized machine learning model, it forecasts the time it would take for data to travel between the user and the execution node.
  (2) NetworkLatency=g(Longitude,Latitude,MLmodel)
  where:
  - *Longitude* and *Latitude* are the geographic coordinates of the user.
  - *MLmodel* signifies the machine learning model dedicated to latency prediction.
  - *g* is the function determining the network latency.
3. **Placement Decision:** The final stage involves synthesizing the outcomes of the previous stages—execution time and network latency predictions and comparing them with the deadline associated with the function. This comprehensive analysis enables the Logic Processor to identify the most suitable node for executing the function within the stipulated deadline.
  (3) NodeSelection=h(ExecutionTime,NetworkLatency,Deadline)
  where:
  - *ExecutionTime* and *NetworkLatency* are the estimates from the first two stages.
  - *Deadline* is the time constraint tied to the function.
  - *h* represents the decision-making function that integrates these parameters to determine the optimal node for function placement.

This structured approach ensures a meticulous assessment, taking into account both computational and network performance to facilitate the efficient execution of functions within their respective deadlines.

### 5.2. Execution Deployment Module

The purpose of this module can be expressed by Algorithm 3 based on the decision made considering execution time prediction, network latency prediction, and the deadline of a task (line 2 of Algorithm 3). The compute node is maintained and filtered to choose the node which can best execute the function within the specified time limit (line 4 of Algorithm 3). When the appropriate compute node is selected for the user the function, execution is triggered and the function is placed on the appropriate node.   
**Algorithm 3:** Function Placement Decision
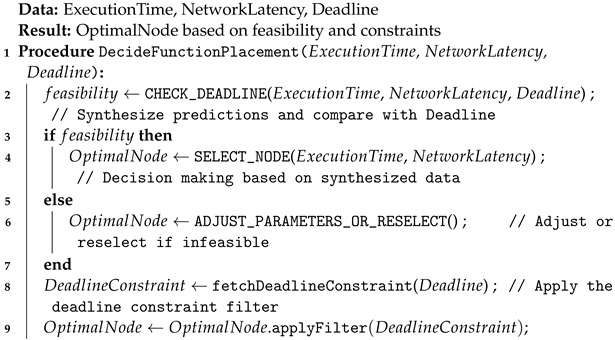


### 5.3. XGBoostRegression

XGBoostRegressor is a popular choice for regression problems due to its effectiveness in handling complex data patterns and its ability to provide accurate predictions.

1. **Ensemble Learning**: XGBoostRegressor is an ensemble learning method that combines the predictions of multiple weak learners (decision trees) to create a strong and robust model. This is particularly advantageous when dealing with a multifaceted problem like function placement in cloud computing, where various factors can influence the outcome.
2. **Gradient Boosting**: XGBoost uses gradient boosting, which is an iterative approach to improving model accuracy. It minimizes the residuals (the differences between the predicted and actual values) in each iteration. This is essential in function placement, as we want to continually refine the predictions based on real-time data and evolving conditions.
3. **Regularization**: XGBoost incorporates regularization techniques such as L1 (Lasso) and L2 (Ridge) regularization. This helps prevent overfitting, which is crucial when dealing with complex datasets and optimizing function placement strategies.

Mathematical Equation:

The objective function that XGBoost seeks to optimize can be expressed as follows:

(4) $$L\left(\theta \right)=\sum_{i=1}^{n}l\left(y_{i},{\hat{y}}_{i}\right)+\sum_{k=1}^{K}\Omega \left(f_{k}\right)$$

This equation outlines the XGBoost objective function, which combines the loss function and regularization term to be minimized during the training process.

In practice, XGBoostRegressor optimizes this objective function through gradient boosting and regularization, iteratively improving the model’s predictions and minimizing the overall loss. This simplified equation demonstrates how XGBoostRegressor combines predictions from multiple decision trees while optimizing the loss function and applying regularization, making it a powerful choice for regression problems, including function placement in cloud computing.

## 6. Evaluation and Results

In order to replicate and maintain the specific environmental conditions accurately, The proposed model extensively utilized Docker containers across the board. This approach ensured that the computational resources were precisely allocated to each node according to a deliberate scheme. For instance, Edge1 received a specific allocation of 0.2 CPU cores and 750 MB of memory, while Edge2 was provisioned with 0.3 CPU cores and 650 MB of memory. On the other hand, cloud nodes were equipped with distinct configurations: one set with three CPU cores and 1.5 GB of memory, and another set with two CPU cores and 1 GB of memory. These intentionally varied memory and CPU core allocations were instrumental in introducing deliberate heterogeneity into the proposed model, a strategic move shaping the core of the experimental setup.

**Workload orchestration:** The choice of application for the experimental scenario revolved around a video surveillance camera system, a domain intricately detailed in [35]. This selection perfectly aligns with the research objectives due to its inherent event-driven nature and stateless characteristics. The surveillance system is tasked with processing high volumes of video data from hundreds of cameras, requiring substantial computational resources. The infrastructure spans both cloud data centers and edge computing devices to ensure timely analysis and response.In summary The evaluation centers on three critical performance metrics: optimizing function placement, QoS (deadline), and network latency.

### 6.1. Optimizing Function Placement

The system utilizes a mix of execution nodes, from powerful servers in the cloud to less capable edge devices near the cameras. The variability in computational power and storage capacity across these nodes affects the efficiency of processing tasks. As the computation power of each node is different, so is their execution power. This is due to the fact that in real-world scenarios, execution nodes are more heterogeneous in nature than homogeneous. The first set of data in Table 2 reflects the average execution time for the face detection function (FD), a computationally intensive task particularly sensitive to CPU allocation. Edge1 and Edge2, with their modest CPU and memory provisions, clocked in at 9.066104 s and 5.422694 s, respectively. In stark contrast, the cloud nodes, with significantly more robust configurations, demonstrate markedly superior performance, with Cloud1 and Cloud2 executing the same task in a mere 0.349498 s and 0.318884 s, respectively. This divergence in execution times starkly illustrates the impact of computational resources on task efficiency, validating the hypothesis that edge nodes are more suited for lightweight tasks, while the cloud is ideal for heavy-duty computations.

Upon examining the gif creation function in Table 2, the trend in average execution times remains consistent, though the absolute figures shift. The edge nodes, particularly Edge1 with its highest time of 26.151723 s, reveal a noticeable delay compared to their cloud counterparts, which maintain a sub-second performance. Notably, Edge2’s enhanced CPU allocation appears to yield dividends, outperforming Edge1 and reinforcing the principle that even slight improvements in resources can significantly affect performance outcomes. The image resizing function, a less CPU-intensive operation, shows a more leveled playing field. Edge1’s time of 0.206138 s and Edge2’s time of 0.058517 s are comparatively closer to the cloud nodes’ times, indicating that certain functions may not necessitate the formidable capabilities of the cloud. This suggests that certain tasks can be efficiently offloaded to the edge without a substantial compromise in performance, offering a strategic opportunity to balance the load across the proposed model’s diverse computational landscape.

Lastly, the video conversion function, another resource-heavy task, demonstrates a stark differentiation in execution times. Edge1 and Edge2 grapple with significantly higher times of 133.720801 s and 88.903799 s, respectively, in contrast to the cloud nodes, which manage to perform the same task in just a few seconds. This emphasizes the role that resource allocation plays in heavier processing tasks and spotlights the cloud’s ability to handle more demanding applications with ease.

### 6.2. Quality of Service (Deadline)

**Figure 4:**
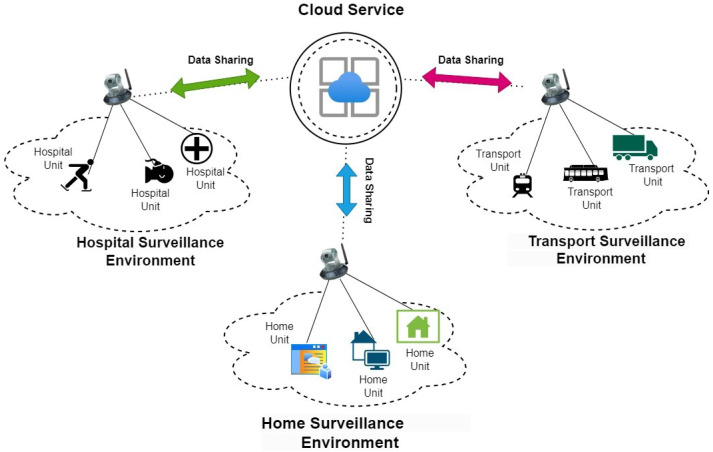
Example of surveillance camera in cloud.

The serverless platform must decide where to execute each analytical function, such as motion detection or object recognition, without specific regard for the urgency of the situation. For instance, analyzing footage for a reported theft in progress demands faster processing compared to routine surveillance. Without considering these task deadlines, the system might delay critical analyses, potentially missing crucial intervention opportunities. Figure 4 gives an idea of how a surveillance camera is depicted in cloud architecture. With every field is based on a QoS, it is important to give timely response. This setup allowed us to orchestrate the workload effectively, an essential pursuit in achieving the research goals. The comparison of function execution between the proposed system and the baseline paper employs functions such as **FD, GC, IR and VC**. The quality of service (**QoS metrics**), crucial for the research, centered around event deadlines, including 5, 3, 1, 8. The images spanned from 100 KB to 2 MB, while video sizes varied from 1 MB to 4 MB, often arriving with varying resolutions and time periods [36].

**Figure 5:**
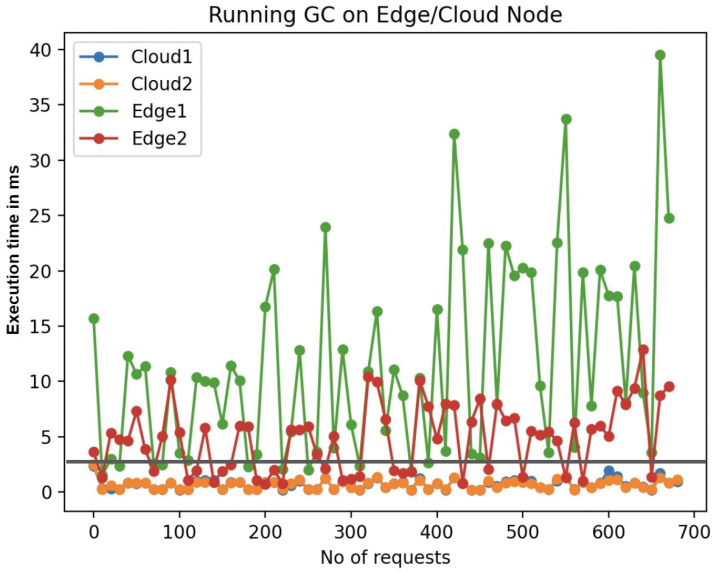
Function execution in cloud and edge.

**Figure 6:**
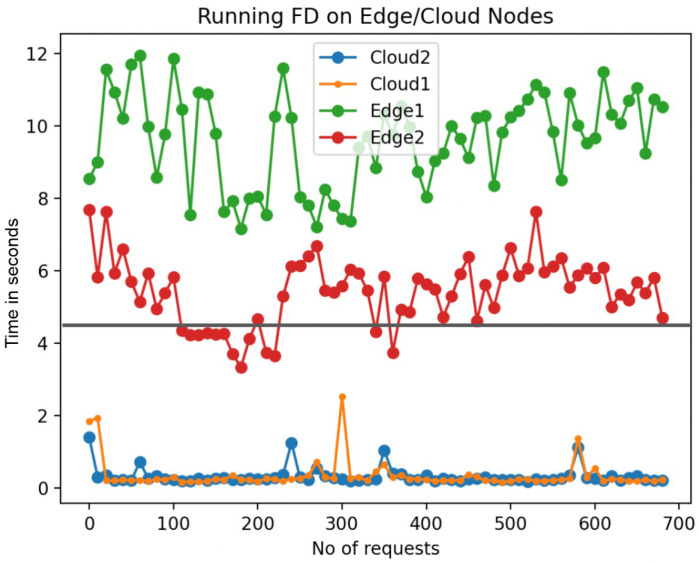
FD Function execution in cloud and edge.

**Figure 7:**
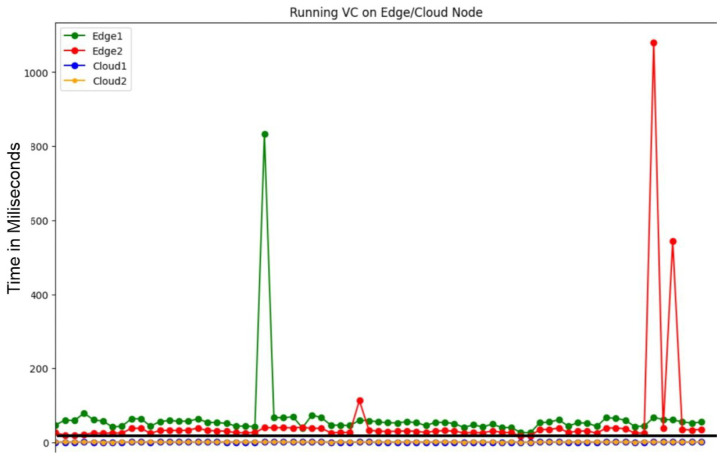
VC Function execution in cloud and edge.

The crux of QoS in a serverless architecture lies in its ability to meet predefined, task-specific deadlines. As shown in Figure 5, Figure 6 and Figure 7, the variability in execution times for face detection (FD), gif creation (GC), and video conversion (VC) elucidates the necessity of judicious function placement.

In gif creation (GC) (Figure 5), with a stricter deadline of 3 s, the edge nodes displayed inconsistency with occasional spikes beyond the acceptable window. This variability could be detrimental in high-stakes scenarios where every second counts. The cloud nodes’ performance remained comfortably within the deadline, asserting their capability to handle such operations without breaching QoS parameters.

For face detection (FD) (Figure 6), the deadline was set at 5 s. The edge nodes, despite their proximity to data sources, could not consistently meet this benchmark. The cloud nodes, however, consistently processed requests under the deadline, showcasing their reliability for time-sensitive tasks.

The video conversion (VC) (Figure 7) task posed an even more substantial challenge with a deadline of 8 s, which the edge nodes failed to meet, underscoring their unsuitability for such heavy workloads. The cloud nodes, while generally maintaining performance within the deadline, displayed rare spikes that could indicate the need for additional optimization to ensure unwavering compliance with QoS demands.

These results solidify the argument that for QoS adherence, especially when deadlines are stringent, the computational capabilities of each node must be factored into function deployment strategies. Cloud nodes, with their superior resources, are the reliable choice for tasks where deadlines are non-negotiable, while edge nodes may suffice for less urgent tasks, offering a balanced and strategic utilization of overall system resources.

The orchestration of workloads, as showcased in Figure 5, Figure 6 and Figure 7, underpins the pursuit of the research goals—optimizing function placement in adherence to event-driven deadlines, ensuring that the system’s response is not only timely but also strategically distributed across the serverless infrastructure to maintain QoS in varying surveillance scenarios. This nuanced approach to function execution aligns with the research’s aim to efficiently manage the computational diversity inherent in a heterogeneous serverless environment.

To train the machine learning model effectively, native functions closely related to the video surveillance domain, including face detection, image resizing, gif creation, and video conversion, were utilized and executed across both edge and cloud nodes. The data generated from these executions formed the backbone of the machine learning model’s training dataset, encompassing crucial information such as resolution, execution time, CPU utilization, and memory consumption.

**Figure 8:**
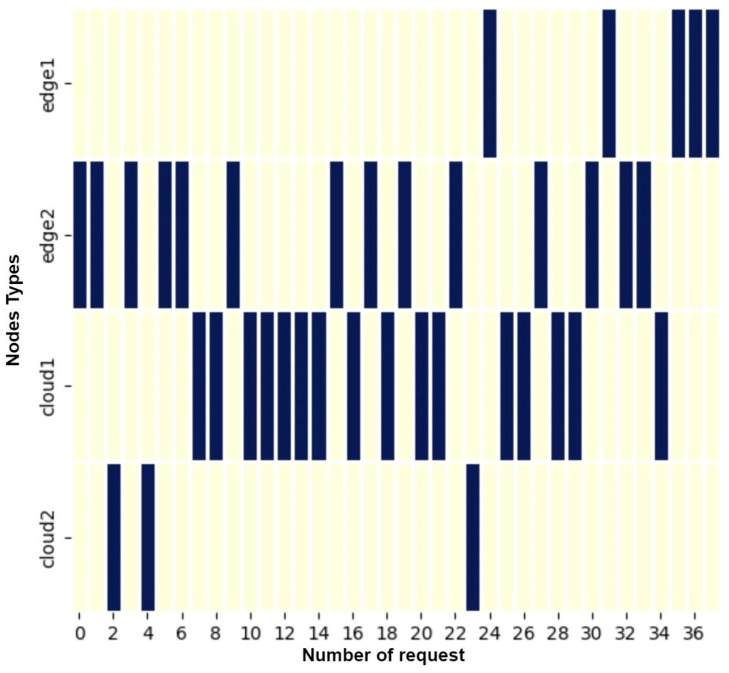
Function placement in cloud and edge nodes.

The machine learning model underwent rigorous training, leveraging an extensive dataset comprising 880 images per function. This comprehensive dataset played a pivotal role in informing the decision-making capabilities of the model, resulting in a remarkable 94 percent accuracy, outshining the baseline paper’s 85 percent accuracy [17]. In Figure 6, the allocation of functions within the heterogeneous infrastructure is demonstrated, revealing a predominant placement within Cloud1. The primary reason behind this is the significantly high execution time for VC (video conversion) on the edges. Placing tasks on either of the edges would likely lead to missed deadlines. Consequently, a majority of placements were made on Cloud1. Tasks requiring additional processing power to meet the deadlines were consequently shifted to Cloud2. Functions were primarily situated in this environment, with a selective migration to Cloud2 necessitated by the inability to meet the QoS requirements within the designated time frame. Only a minimal subset of functions was deployed across both edge environments, a strategic decision made due to latency constraints that could potentially compromise user QoS expectations. Similarly, in Figure 8, function GC placement is also shown among the heterogeneous setup. The request comes with varying workloads and different resolutions. The blue lines indicate the requests executed on a specific node, while the yellow lines indicate those not executed on this node. The system places the functions on the execution nodes. As the system adapts and places the functions according to the deadlines, it intelligently distributes the load among the edges and clouds to meet the deadline and not saturate one node. The X-axis represents the number of requests, and the Y-axis represents the execution nodes types, which are cloud1, cloud2, edge1, and edge2.

### 6.3. Network Latency

**Figure 9:**
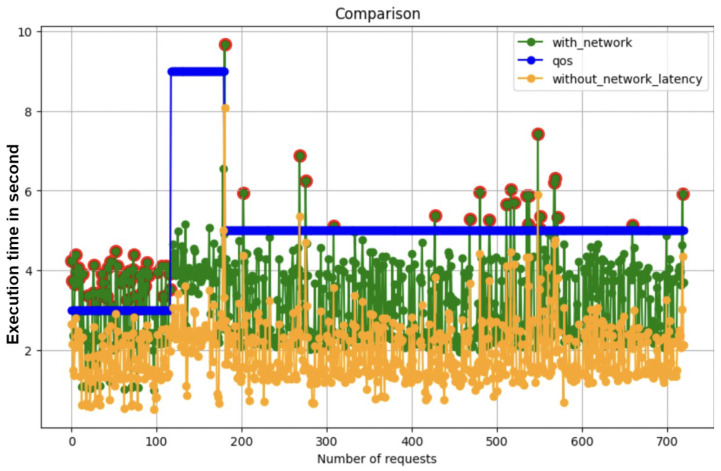
Function placement with network latency consideration and without.

One significant aspect of the model was its network latency predictive functionality. Designed to predict network latency based on geographical coordinates (longitude and latitude) inputted into the model, this feature assumed immense significance. As depicted in Figure 9, meeting deadlines emerged as an absolute priority. Initial observations without considering network latency consistently met deadlines. This provides an essential insight into the role of network latency in function placement within serverless architectures. This illustrates the significant difference in deadline compliance when network latency is considered versus when it is not.

- **Observation without Network Latency**: Initially, the function placement strategy, without considering network latency, showed a high rate of success in meeting deadlines. This observation might give an impression of efficiency, but it overlooks a critical component of real-world deployments—network latency. It represents the baseline placement decision when network latency was not considered in the system.
- **Impact of Integrating Network Latency**: The introduction of network latency into the execution time calculations brought a paradigm shift. The baseline approach, which previously showed high success rates, now struggled to meet deadlines. This stark contrast highlights the profound impact of network latency on function placement decisions. It represents the the proposed system capability to not only predict the network latency but also make placement decision to meet the deadline of the task.
- **Role of Network Latency in Strategy**: These findings underscore the pivotal role of network latency in formulating an effective function placement strategy. It is a clear indication that any practical approach must incorporate latency considerations to ensure reliability and efficiency in real-world scenarios.
- **Model Accuracy**: For the prediction of network latency, a decision tree regressor was employed, achieving approximately 70% accuracy. This accuracy level represents a significant step towards understanding and integrating latency impacts into serverless computing models.

Given our proposed system, the equation for network latency consideration can be derived as:

- **Function Execution Time (FET)**: This is the time taken by a function to complete its task without considering network latency.
- **Network Latency (NL)**: The time delay introduced due to the network. This includes both the time for a request to travel from the client to the server (latency) and the time for the response to travel back (response time).
- **Total Execution Time (TET)**: This is the overall time taken for a function to execute, including the network latency.

Given these definitions, we can model the Total Execution Time as the sum of the Function Execution Time and the Network Latency. The equation can be represented as:

(5) TET=FET+NL

Considering and implementing all the parameters, the proposed system was not only able to meet maximum deadlines but also to adapt function placement decisions according to changing deadlines and workload, as depicted in Figure 6.

To further explore that ML models are a better fit for function placement and meeting deadlines, the research further compares the proposed architecture with non-ML algorithms for function placement:

1. Best Fit
2. Round Robin

**Figure 10:**
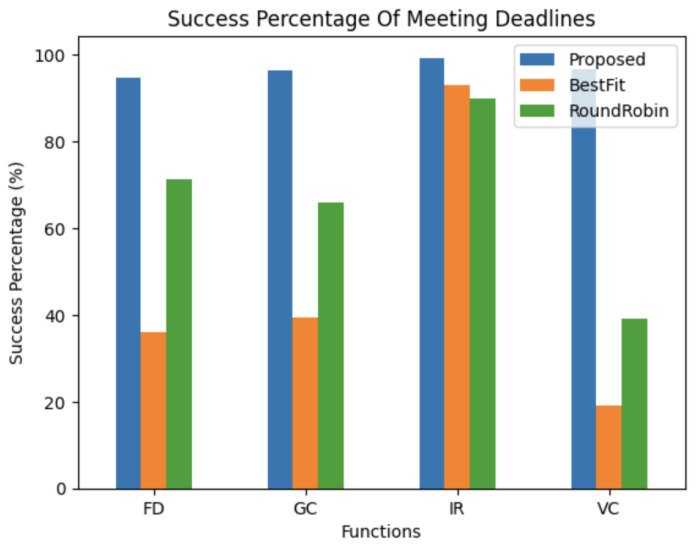
Deadline success rate between proposed method and BestFit.

**Best Fit**: The method functions based on the fundamental premise of maximizing function distribution among computer nodes while minimizing resource consumption [17]. Within the framework of the setup, the Best Fit algorithm attempts to assign functions mostly to edge nodes prior to using cloud resources. From a logical standpoint, as well as from the graphic depiction Figure 10, it is clear that the Best Fit algorithm has more missed deadlines than the proposed approach. This discrepancy results from the Best Fit algorithm’s failure to take deadlines into account when determining where to position functions.

**Figure 11:**
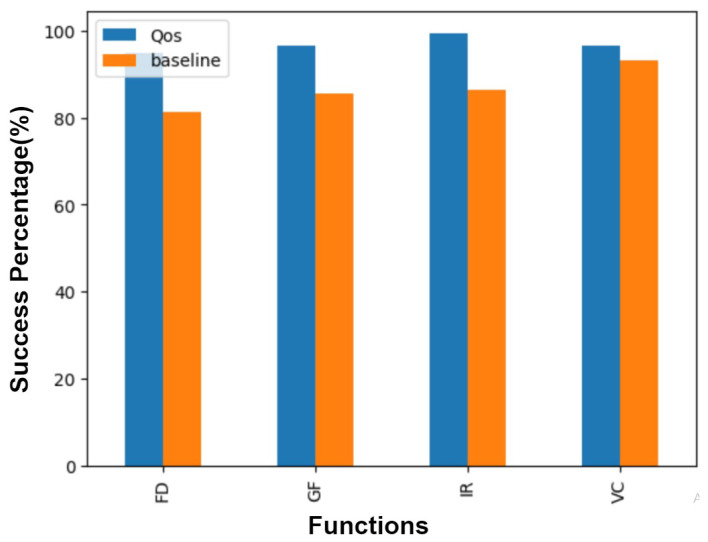
Success ratio between proposed method and the baseline paper.

An essential metric for evaluating the effectiveness of our proposed machine learning model for function placement is its ability to meet operational deadlines, especially when compared to existing approaches. Figure 11 illustrates the performance of our system in comparison to a baseline system described in prior research. The blue bars represent the number of deadlines met by our proposed system for four distinct functions, while the orange bars depict the performance of the baseline system.

As shown in Figure 11, our system consistently meets a higher number of deadlines across all functions tested. This improvement underscores the efficacy of our ML-driven approach in accurately predicting and optimizing function placements in a serverless computing environment.

**Round Robin**: In earlier studies, the Round Robin scheduling technique was frequently used to handle function placement. In order to avoid oversaturation, Round Robin distributes the computational load among nodes cyclically [28]. Although this method seems advantageous in theory, the fact that deadlines are attached to events highlights how crucial it is to take the capacity of the individual compute nodes into account.

Round Robin increases the probability of missing deadlines by regularly distributing the load equally among all nodes, as seen in Figure 10. In contrast, compared to Round Robin, the suggested model—which takes node capabilities into account—shows noticeably fewer missed deadlines.

The comparison highlights not only the practical benefits of our approach in reducing latency and improving operational efficiency but also its robustness in maintaining service-level agreements (SLAs) under diverse conditions. The ability to meet deadlines more reliably than the baseline system demonstrates the potential of our proposed solution to address some of the most pressing challenges faced by developers and cloud service providers in a serverless architecture.

This significant improvement in deadline adherence can be attributed to several key factors. First, the machine learning model’s capacity to learn from historical data allows for more accurate predictions of function execution time, considering factors such as network latency and resource availability. Second, by optimizing function placement closer to the data source or within more capable compute nodes, our system minimizes the delays often encountered in traditional cloud or edge computing scenarios. Lastly, the dynamic scaling capabilities of our serverless architecture ensure that resources are efficiently allocated in response to real-time demand, further contributing to our system’s superior performance.

Table 3 summarizes our contribution and the consideration that we have taken to develop our system, so the scenarios could be as close to the real world as possible, employing parameters that depict the real-world challenges.The results show that our suggested method is scalable because it intelligently distributes workloads across different nodes in a heterogeneous infrastructure, ensuring that tasks fulfill deadlines without overloading any single node. The machine learning model, which achieved 94 percent accuracy, heavily informs this dynamic allocation procedure.The following points demonstrate scalability:

1. Dynamic Allocation: The system assigns functions according to their execution time and workload needs. The system routes tasks with long execution periods that could miss deadlines on edge nodes to cloud nodes, ensuring their timely completion. This dynamic allocation keeps a single node from becoming a bottleneck.
2. Load Balancing: The system responds to changing workloads and resolutions by dividing the load among the available nodes (Cloud1, Cloud2, Edge1, and Edge2). This helps to maintain equilibrium by preventing any single node from becoming saturated, which is critical for scalability.
3. Selective Migration: The system migrates functions between nodes, such as Cloud1 and Cloud2, according to their processing power and quality of service (QoS) requirements. This selective migration aids in the efficient management of resources, allowing the system to accommodate an increasing number of requests while maintaining performance.

## 7. Conclusions and Future Work

This paper’s primary focus is on optimizing the deployment of serverless applications, which are comprised of multiple serverless functions, within both edge and cloud computing environments considering the heterogeneity of the nodes. The central goal is to deliver high-quality services to a large number of connected users. To achieve this objective, this paper predicts the allocation of functions between cloud and edge nodes based on the QoS requirements and users’ geographical positions.To make these predictions, this paper employs a non-linear machine learning model, XGboostRegressor, which has been trained using data related to function execution time, CPU usage, memory consumption, and image resolution. The performance of the proposed deployment strategy is evaluated, and the results indicate its effectiveness in function placement. In the future, merging serverless computing with blockchain technologies such as Function as a Service (FaaS) has the potential to significantly improve scalability and efficiency. Organizations can achieve dynamic scalability by incorporating FaaS into blockchain frameworks such as smart contracts or decentralized apps (DApps). FaaS allows for real-time automatic scaling of computer resources, maximizing resource utilization and lowering operational costs. This integration enables blockchain networks to handle higher transaction volumes, more complicated computations, and a wider range of use cases without requiring manual infrastructure provisioning or management. Furthermore, FaaS in blockchain systems encourages shorter development cycles, faster deployment of decentralized solutions, and greater agility in responding to changing business needs and market dynamics. 

## Figures and Tables

**Table 1:** Summary of identified research gaps through available literature. Tick = includes; X = does not include.

Ref. No.	Greedy/Heuritic	Network Latency	Heterogeneity	Deadline Consideration	Machine Learning
[4]	✓	X	✓	X	X
[15]	✓	X	✓	X	X
[25]	✓	✓	X	X	X
[26]	✓	X	X	✓	X
[27]	X	✓	X	✓	X
[28]	✓	X	X	✓	X
[17]	✓	✓	X	X	X
[29]	✓	X	X	✓	X
[30]	✓	X	✓	✓	X
[7]	X	✓	X	✓	✓
[31]	✓	✓	X	✓	X
[34]	✓	✓	✓	✓	X
[33]	✓	✓	X	✓	X

**Table 2:** Average execution time in cloud and edge.

Server	FD Average Execution Time (s)	GC Average Execution Time (s)	IR Average Execution Time (s)	VC Average Execution Time (s)
Edge1	9.06	26.15	0.206	133.72
Edge2	5.42	4.6	0.05	88.93
Cloud1	0.34	0.67	0.053	2.49
Cloud2	0.31	0.62	0.52	1.176

**Table 3:** Comparison between proposed methodology and previous contributions to the field and limitations. Tick = includes; X = does not include.

Ref. No.	Network Optimization	Heterogeneity	Deadline Optimization	ML Driven	Algorithm Approach	Edge-Cloud Continuum
[4]	X	✓	X	X	Heuristic	✓
[15]	X	✓	X	X	Heuristic	X
[25]	✓	X	X	X	Heuristic	✓
[26]	X	X	✓	X	Heuristic	X
[27]	X	X	✓	X	Greedy	X
[28]	✓	X	X	X	Greedy/Heuristic	✓
[17]	✓	X	X	X	Heuristic	X
[37]	X	✓	✓	✓	Linear Regression	✓
Proposed	✓	✓	✓	✓	XGboostRegressor	✓

Note: Comparison based on the research areas of the serverless method.

## Data Availability

Data is contained within the article.
